# Performance of Three Mortality Prediction Scores and Evaluation of Important Determinants in Eight Pediatric Intensive Care Units in China

**DOI:** 10.3389/fped.2020.00522

**Published:** 2020-09-08

**Authors:** Zhengzheng Zhang, Xiangyuan Huang, Ying Wang, Ying Li, Hongjun Miao, Chenmei Zhang, Guoquan Pan, Yucai Zhang, Xiaodong Zhu, Weiming Chen, Juanzhen Li, Dongni Su, Yanlong Bi, Zhenjie Chen, Bingxin Jin, Huijie Miao, Xiangmei Kong, Ye Cheng, Yang Chen, Gangfeng Yan, Weili Yan, Guoping Lu

**Affiliations:** ^1^Pediatric Emergency and Critical Care Center, Children's Hospital of Fudan University, Shanghai, China; ^2^Department of Clinical Epidemiology, Children's Hospital of Fudan University, Shanghai, China; ^3^Pediatric Intensive Care Unit, Shanghai Children's Medical Center, Shanghai Jiaotong University School of Medicine, Shanghai, China; ^4^Intensive Care Unit, Children's Hospital of Soochow University, Suzhou, China; ^5^Department of Emergency, Children's Hospital of Nanjing Medical University, Nanjing, China; ^6^Pediatric Intensive Care Unit, Children's Hospital of Zhejiang University School of Medicine, Hangzhou, China; ^7^Pediatric Intensive Care Unit, The Second Affiliated Hospital & Yuying Children's Hospital, Wenzhou Medical University, Wenzhou, China; ^8^Department of Critical Care Medicine, Shanghai Children's Hospital, Shanghai Jiao Tong University, Shanghai, China; ^9^Department of Pediatric Critical Care Medicine, Xinhua Hospital Affiliated to Shanghai Jiaotong University School of Medicine, Shanghai, China

**Keywords:** pediatric intensive care unit, mortality, cohort study, prediction model, model validation

## Abstract

**Background:** The mortality prediction scores were widely used in pediatric intensive care units. However, their performances were unclear in Chinese patients and there were also no reports based on large sample sizes in China. This study aims to evaluate the performances of three existing severity assessment scores in predicting PICU mortality and to identify important determinants.

**Methods:** This prospective observational cohort study was carried out in eight multidisciplinary, tertiary-care PICUs of teaching hospitals in China. All eligible patients admitted to the PICUs between Aug 1, 2016, and Jul 31, 2017, were consecutively enrolled, among whom 3,957 were included for analysis. We calculated PCIS, PRISM IV, and PELOD-2 scores based on patient data collected in the first 24 h after PICU admission. The in-hospital mortality was defined as all-cause death within 3 months after admission. The discrimination of mortality was assessed using the area under the receiver-operating characteristics curve (AUC) and calibrated using the Hosmer–Lemeshow goodness-of-fit test.

**Results:** A total of 4,770 eligible patients were recruited (median age 18.2 months, overall mortality rate 4.7%, median length of PICU stay 6 days), and 3,957 participants were included in the analysis. The AUC (95% confidence intervals, CI) were 0.74 (0.71–0.78), 0.76 (0.73–0.80), and 0.80 (0.77–0.83) for PCIS, PRISM IV, and PELOD-2, respectively. The Hosmer–Lemeshow test gave a chi-square of 3.16 for PCIS, 2.16 for PRISM IV and 4.81 for PELOD-2 (*p* ≥ 0.19). Cox regression identified five predictors from the items of scores better associated with higher death risk, with a C-index of 0.83 (95%CI 0.79–0.86), including higher platelet (HR = 1.85, 95% CI 1.59–2.16), invasive ventilation (HR = 1.40, 1.26–1.55), pupillary light reflex (HR = 1.31, 95% CI 1.22–1.42) scores, lower pH (HR 0.89, 0.84–0.94), and extreme PaO_2_ (HR 2.60, 95% CI 1.61–4.19 for the 1st quantile vs. 4th quantile) scores.

**Conclusions:** Performances of the three scores in predicting PICU mortality are comparable, and five predictors were identified with better prediction to PICU mortality in Chinese patients.

## Introduction

Patients in pediatric intensive care units (PICU) always have a higher risk of death. The PICU mortality rate in China is two or three times that of developed countries in America and Europe (1–4). It is very important to identify predictors or determinants of death in PICU. Since the establishment of PICU, critical care researchers have been constantly exploring the death risk prediction scores. At present, the most widely used scores in PICUs are PRISM III/IV (5, 6), PIM3 (7) and PELOD-2 (8), but their performances and comparisons in Chinese PICU patients in large sample sizes have not been reported. The Pediatric Critical Illness Score (9) (PCIS) has been commonly used in China for the severity assessment of PICU patients. It was established in 1995 based on Chinese experts' experience and only available in Chinese version for domestic use (translated PCIS scale can be seen in Supplementary Table 1).

The performances of PRISM and PIM applied in PICU patients have been assessed earlier in Hong Kong, showing good predictive accuracy with area under the receiver operating characteristic curve (AUC) over 0.9 (10, 11). However, studies from mainland Chinese patients were limited, and most of them were based on single-center samples (12–14), partly due to the unavailability of required data and facilities of the international scores in China. Compared with the reports in Americans and Europeans, these studies showed less ideal performances of these scores in predicting PICU mortality in Chinese patients; the AUCs were 0.73–0.83 for PRISM, 0.72–0.75 for PIM, 0.77 for PELOD-2, and 0.64 for PCIS. The situations are unknown in a larger number of Chinese patients. The aim of the current study is to evaluate the performances of PCIS, PRISM IV, and PELOD-2 in PICU mortality prediction based on a large multicenter cohort of Chinese patients, and to explore the possibility of identifying a smaller number of important determinants to mortality, which are more easily acquired in most PICUs of China.

## Materials and Methods

### Study Design and Participants

This was a multicenter prospective observational cohort study including eight PICUs of tertiary teaching hospitals with similar organizations, staffing structures, and management protocols in China; four in Shanghai, two in Jiangsu Province, and two in Zhejiang Province. These eight hospitals are located in the prosperous Yangtze River Delta region and represent more than medium-level PICUs in China. Patients who were admitted to these PICUs between Aug 1, 2016, and Jul 31, 2017, and met the following criteria were eligible and recruited consecutively: a) age over 28 days and below 18 years on admission (the same with patients' admission criteria of PICU in China) and b) patients staying over 4 h in PICU (to minimize missing data). Readmissions to the PICU during the same hospitalization were recorded as two admissions. No interventions or procedures beyond routine clinical practice were implemented. This study was reviewed and approved by the Institutional Review Board (IRB) at the Children's Hospital of Fudan University. Guardians of all participants were informed and signed a routine consent form on admission including the future use of their data for research purposes. No consent form specific to this study was signed. The study protocol was registered at Clinicaltrial.gov NCT02961153.

### Data Collection

A uniform case report form was developed for prospective data collection, including hospital facilities of PICU, demographics, clinical, laboratory and therapeutic data of patients. Demographic data included age, gender, date of birth, and payment type. Clinical data included admission diagnosis classified by the system of primary dysfunction based on reason for admission (international classification of disease, ICD-9), etiology of diseases (infections, poisonings, accidents, immunity, tumors, congenital malformations, metabolic disorders, and others), admission sources (general wards, emergency departments, outpatients, operating rooms, transferred from another hospitals), status at the time of admission [cardiopulmonary resuscitation performed 24 h before PICU admission, PICU hospitalization previously associated with this admission, 24 h after surgery but not including postanesthetic recovery patients (those patients were admitted to PICU because of the difficulty in getting beds), invasive ventilator support, vasopressors support], and underlying diseases (chronic health status in the last 3 months before PICU admission), dynamic vital signs (temperature, heart rate, respiratory rate, systolic and diastolic blood pressures) from the time of admission to 24 h after admission, and pupillary reactions and Glasgow Coma Score (included only patients with central nervous system diseases). Laboratory data included blood gases (pH, PCO_2_, bicarbonate, total CO_2_, arterial PaO_2_, lactate), chemistry tests (alanine aminotransferases, aspartate aminotransferases, total and direct bilirubin, albumin, creatinine, urea nitrogen, glucose, serum potassium, and serum sodium), and hematology tests (white blood cell count, hemoglobin, platelet count, prothrombin time or partial thromboplastin time, international normalized ratio, and fibrinogen). PaO_2_ data were obtained from ventilator patients (~24%) and other patients who had arterial blood gas. A small proportion of PaO_2_ data came from venous or capillary samples (~10% of included patients) due to arterial puncture failure. The data of PaO_2_ were analyzed uniformly and some speculations were made. Therapeutic data included a fraction of inspired oxygen, whether treated with invasive ventilator support, vasopressor support (dopamine, dobutamine, epinephrine, or norepinephrine), or continuous blood purification within the first 24 h of admission. Items were measured only if the doctor thought it was appropriate. If it was not measured, it was assumed that the value of the variable was normal or identical to the previous measurement. PRISM IV, PELOD-2, and PCIS were calculated as reported.

### Outcomes

The primary outcome of this study was in-hospital mortality (defined as all-cause death within 3 months after admission). Patients who still survived or transferred to general wards were defined as survival (coded as 0). Patients discharged against medical suggestions were excluded from the analysis since their outcomes were unknown, and the association analysis would be biased if they were included.

Patients were routinely transferred to the general ward when the following criteria were met: effectively control of primary disease, away from mechanical ventilation, blood purification, vasoactive drugs for more than 48 h, or stable vital signs. Transfer to the general ward was confirmed by the PICU attending doctor or senior doctors.

### Quality Control

A database was developed using Microsoft ACCESS based on the uniform case report form, where data can be automatically checked. Two investigators at each site were trained to collect, check, and enter the records. The coordination center monitored the database for quality control; data manager checked with the site about queries with phone or networking applications. Each center recorded the recruitment and reported the number of those discharged against medical suggestions.

### Statistical Analysis

Characteristics were summarized for all patients. Count variables were summarized in count and percentages, and numerical variables were summarized in median and interquartile range (IQR).

The three scores were calculated for patients who did not discharge, and their abilities to discriminate mortality were presented in ROC. Pair-wise comparisons were applied to test differences between AUCs of the three scores. For sensitivity analysis, Cox regression models were fitted and C-indices were calculated and compared among the three scores. Bonferroni adjustment was applied for multiple tests.

Hosmer–Lemeshow goodness-of-fit test was applied to examine the extent to which observed and predicted risks of death agree within quintiles of death risk. The statistic χ^2^ was calculated as a summary indicator of calibration. A higher χ^2^ means greater discrepancy between observed and predicted risks of death.

To identify significant predictors for death, a Cox regression model was constructed. Items from the three score systems were sequentially incorporated, starting from a univariate model with the highest C-index.

All statistical analyses were conducted in STATA 15.0 (Stata Statistical Software, Stata Corp, College Station, TX), and α as the threshold for statistical tests was set at 0.05.

## Results

**Figure 1:**
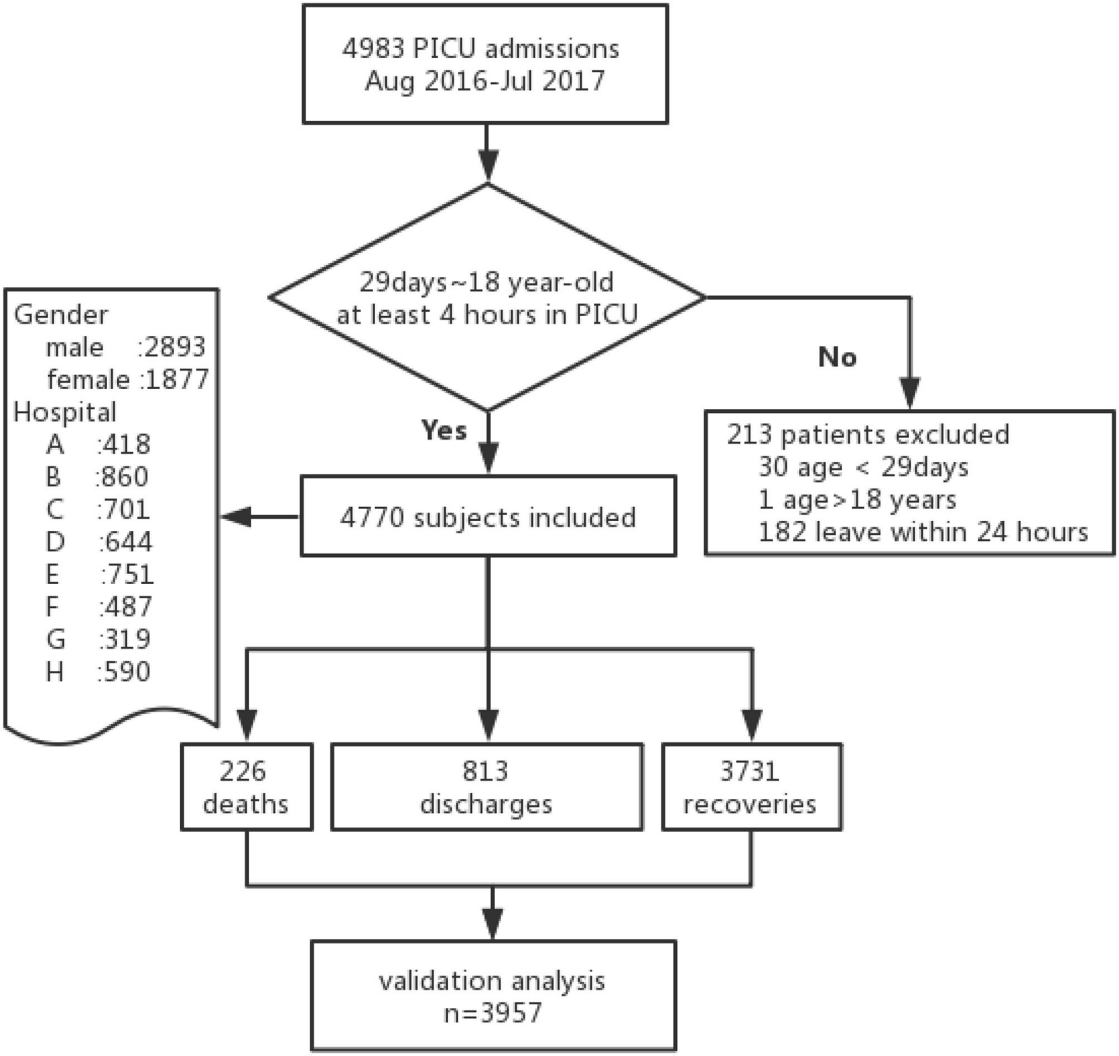
Flow diagram.

A total of 4,770 eligible patients out of 4,983 admissions were recruited in the eight PICU centers during the study period (Figure 1). Two hundred and twenty six patients died in hospital and overall mortality was 4.7%. Three thousand seven hundred and thirty one patients improved and were transferred to the general ward; 813 were discharged against medical suggestions. Of the 4,770 subjects enrolled, 3,957 who were not discharged were included in the analysis.

As in Table 1, 2,023 (42.4%) patients were younger than 1 year old, and mortality rate was similar among age groups (chi-square test *p* = 0.82). 2,893 (60.6%) patients were boys. The median length of PICU stay was 6 days. Contributions of each PICU to the total sample ranged from 6.7 to 18.0%. 2,698 (56.6%) were diagnosed as infection at admission, among whom 116 died. Dead patients had worse risk scores (lower PCIS and higher PRISM III and PELOD-2).

**Table 1:** Characteristics of patients.

**Characteristics**	**Patients *N* = 4,770**	**Deaths *N* = 226**	**Recovery *N* = 3,731**	**Discharge *N* = 813**
**Age**, ***n*** **(%)**				
<1 year	2023 (42.4)	96 (42.5)	1573 (42.2)	354 (43.5)
<3 years	962 (20.2)	44 (19.5)	759 (20.3)	159 (19.6)
<6 years	725 (15.2)	38 (16.8)	570 (15.3)	117 (14.4)
<12 years	773 (16.2)	32 (14.2)	607 (16.3)	134 (16.5)
≥12 years	287 (6.0)	16 (7.1)	222 (6.0)	49 (6.0)
**Gender**, ***n*** **(%)**				
Male	2893 (60.6)	130 (57.5)	2288 (61.3)	475 (58.4)
Female	1877 (39.4)	96 (42.5)	1443 (38.7)	338 (41.6)
**Payment type**^**a**^, ***n*** **(%)**				
Insurance	1993 (42.9)	139 (65.0)	1567 (43.0)	287 (36.5)
No expense	2651 (57.1)	75 (35.0)	2077 (57.0)	499 (63.5)
**Cause of disease**, ***n*** **(%)**				
Infection	2698 (56.6)	117 (51.8)	2173 (58.2)	409 (50.3)
Local infection	2071 (76.8)	58 (50.0)	1729 (80.0)	284 (69.4)
Sepsis	449 (16.6)	31 (26.4)	340 (15.6)	78 (19.1)
Severe sepsis or septic shock	166 (6.2)	27 (23.1)	92 (4.2)	47 (11.5)
Accident	471 (9.9)	31 (13.7)	375 (10.0)	65 (8.0)
Immunology	230 (4.8)	8 (3.5)	182 (4.9)	40 (4.9)
Oncology	282 (5.9)	23 (10.2)	175 (4.7)	84 (10.3)
Congenital malformation	248 (5.2)	17 (7.5)	173 (4.6)	58 (7.1)
Other	843 (17.7)	38 (16.8)	639 (17.1)	166 (20.4)
**Severity Scores, median (IQR)**				
PCIS	90 (86–96)	84 (78–90)	92 (86–96)	88 (82–92)
PELOD-2	2 (1–5)	8 (4–13)	2 (0–4)	4 (2–7)
PRISM IV	5 (2–9)	11 (6–19)	5 (2–8)	7 (3–12)
**Invasive ventilator**, ***n*** **(%)**	1162 (24.4)	152 (67.3)	699 (18.7)	311 (38.3)
**Hypotension**, ***n*** **(%)**	371 (7.8)	54 (23.9)	214 (5.7)	103 (12.7)
**No. co-morbidity**, ***n*** **(%)**				
0	3150 (66.0)	143 (63.3)	2560 (68.6)	447 (55.0)
1	1230 (25.8)	52 (23.0)	922 (24.7)	256 (31.5)
2	341 (7.2)	27 (12.0)	224 (6.0)	90 (11.1)
3 or more	49 (1.0)	4 (1.8)	25 (0.7)	20 (2.5)
**Outcome**, ***n*** **(%)**				
Death in PICU	226 (4.7)			
Recovery	3731 (78.2)			
Discharge	813 (17.0)			
**Time in PICU, day, median (IQR)**	6 (3–12)	6 (2–15)	6 (3–11)	5 (2–13)

a *Payment type missing for 126 subjects; origin missing for 26 subjects*.

*Category variables were summarized with count and percentages, and numerical variables were summarized with median and IQR*.

AUC for the scores' discrimination of mortality were, respectively, 0.76 (95% CI: 0.73, 0.80), 0.80 (95% CI: 0.77, 0.83), and 0.74 (95% CI: 0.71, 0.78) for PRISM IV, PELOD-2, and PCIS as shown in Table 2. After Bonferroni adjustment, AUC for PCIS was significantly lower than that for PELOD-2 (*p* = 0.004) in pair-wise comparisons, while the AUCs for PRISM IV was neither lower than PELOD-2 (*p* = 0.06) nor significantly higher than PCIS (*p* = 0.57). In sensitivity analysis (Supplementary Table 2), C-indices were, respectively 0.79 (0.75, 0.83), 0.81 (0.77, 0.85), 0.77 (0.73, 0.81) for PELOD-2, PRISM IV and PCIS, and pair-wise comparison showed significant difference between PRISM IV and PCIS (*p* = 0.02) only.

**Table 2:** AUC of the scores for predicting risk of death.

**Scores**	**AUC**	**95% CI of hazard ratio**	
		**Lower limit**	**Lower limit**
PRISM IV	0.76	0.73	0.80
PELOD-2	0.80	0.77	0.83
PCIS^*^	0.74	0.71	0.78

* *Significantly lower than PELOD-2*.

As in Table 3, Hosmer–Lemeshow goodness-of-fit test showed good calibration for all three scores (PRISM IV: χ^2^ = 2.16, *p* = 0.54; PELOD-2: χ^2^ = 4.81, *p* = 0.19; and PCIS: χ^2^ = 3.16, *p* = 0.37). As in the Cox regression model (Supplementary Table 3), higher invasive ventilation (HR 1.40, 95% CI 1.26, 1.55), platelet (HR 1.85, 95% CI 1.59, 2.16), and pupillary light reflex (HR 1.31, 95% CI 1.22, 1.42) scores were associated with higher death risk while higher pH (HR 0.89, 95% CI 0.84–0.94) was associated with lower risk of death. PaO_2_ was converted into quantiles for its U-shaped association with death risk, The 1st quantile has the highest mortality risk (HR 2.60, 95% CI 1.61–4.19 compared with the 4th quantile scores). C-index of this model was 0.83 (95%CI 0.79–0.86).

**Table 3:** Results of calibration and Hosmer-Lemeshow test.

**Score**	**Quintiles of risk**	**Observed death**	**Expected death**	**Total**
PELOD-2	1	15	13.8	960
χ^2^ = 4.81	2	20	28.6	1,211
*p* = 0.19	3	16	14.7	435
	4	38	30.6	607
	5	137	138.3	744
	Sum	226	226	3,957
PRISM IV	1	16	15.2	855
χ^2^ = 2.16	2	14	18.2	740
*p* = 0.54	3	32	32.8	940
	4	40	34.1	647
	5	124	125.7	775
	Sum	226	226	3,957
PCIS	1	25	19.8	1,133
χ^2^ = 3.16	2	22	27.1	879
*p* = 0.37	3	14	17.4	416
	4	58	57.4	913
	5	107	104.3	616
	Sum	203	203.2	3,957

Outcome by the hospital can be seen in Table 4. Mortality rates varied among hospitals, which ranged from 2.2% (hospital F) to 5.3% (hospital G). However, hospital C had by far the highest death rate at 16.7%, which was significantly higher than hospital A.

**Table 4:** Outcomes by hospital.

**Center**	**Death**	**Recovery**	**Death rate (%)**	**OR (95% CI)^**a**^**	***P*-value**
A	18	380	4.5	1	/
B	30	678	4.2	0.93 (0.51–1.70)	0.82
C	94	469	16.7	4.23 (2.51–7.13)	<0.001
D	18	464	3.7	0.82 (0.42–1.60)	0.56
E	19	635	2.9	0.63 (0.33–1.22)	0.17
F	9	395	2.2	0.48 (0.21–1.08)	0.08
G	13	233	5.3	1.18 (0.57–2.45)	0.66
H	26	477	5.2	1.11 (0.59–2.06)	0.75

a *Hospital A as reference*.

## Discussion

This study has compared the performances of three scores (PCIS, PRISM IV, and PELOD-2) in predicting the risk of death in Chinese PICU patients based on a large sample size. We found that performances of the three scores are comparable but less satisfying compared with reports from previous studies in Americans and Europeans (15–18). Five items from the scores were identified with better mortality prediction in Chinese patients, namely, platelet, invasive ventilation, pupillary light reflex, pH, and PO_2_.

This study was the first multi-center epidemiological survey on PICU death in China, including eight PICUs from the Yangtze River Delta region, which represented the highest level of PICUs in the country. The overall mortality rate was 4.7%, which was significantly lower than the high mortality in the early days of PICU (12.8%) (19); but it was still at least twice that of US/European PICUs, which had a mortality rate of about 2.5% (2, 3). The causes of the higher mortality in Chinese PICUs were multifactorial. Firstly, the population in this study came from eight centers that received the most serious patients referred from local hospitals routinely. Secondly, there were some differences in the patients' characteristics of PICU patients in our study from studies in western countries. For example, severe pneumonia still ranked as the first cause of death, and one-third of the patients dying of severe pneumonia had underlying diseases, including congenital heart disease, primary immunodeficiency disease, neuromuscular disease, and congenital genetic metabolic diseases, and treatments were more difficult for these patients. In addition, the common application of broad-spectrum antibiotics in order to achieve timely control of bacterial infections may increase the risk of pan-resistant bacteria, super bacteria, and double infections, which were important causes of mortality. Thirdly, higher mortality in the PICUs included in our study was largely attributed to the underdeveloped medical care levels and qualities in China. Compared with developed countries, there was still a large gap in medical care quality which needed great effort to improve.

PRISM and PELOD scores were widely used internationally and showed excellent performance where the scores were developed, with AUCs close to 1 (5, 6, 8). However, their performances were less ideal in this study in a current study sample. One possible explanation could be the different characteristics of the study population. For example, more than half of the cases admitted to PICU were due to respiratory infection in our study, indicating different characteristics of patients (types of diseases), which is much higher than western countries (3, 20). Additionally, the mortality risk varied among countries and hospitals, which was likely attributed to a different quality of PICU care and clinical settings (21). The AUC of this study was similar to a Pakistan study (22) (AUC was 0.78 for PRISM, 0.77 for PELOD), in which earlier versions of the scores were based on a single center and small sample size.

We found an inferior performance of PCIS that was inferior to PRISM IV or PELOD-2. Compared with evidenced-based PRISM IV and PELOD-2, PCIS was established based on experts' experience and lacked scientific evidence. It was older and the predictors of the score were never updated in time with the changes in clinical monitoring indicators. To further explore better predictors of mortality among most recent patient cohort, this study scrambled all the variables of the three scores and analyzed each of them. Five predictors were significantly associated with higher death risk, only two of which were included in the PCIS score. The rest of the three items, invasive ventilation, platelet, and pupillary light reflex, were quite consistent with the characteristics of the patient cohort, where severe respiratory infections, neuromuscular disease, accident, and hematogenous tumor comprised the most common diagnoses on admission. The five identified predictors in the current study improved the performance of the scores; however, they needed to be verified with additional independent study samples. More attention was expected in updating PCIS.

This study has the following strengths in methodology. Firstly, this was a multicenter study with the largest sample size of PICU patients in China, making the findings more generalizable. Secondly, this study was prospectively designed and carried out; possible predictors were deliberately collected with quality control procedures, making the data more reliable.

## Limitations

A total of 813 eligible patients were discharged against medical suggestions, whose outcome could not be observed. The reasons these patients left hospital may include poor prognosis, low quality of life, and religious belief. It poses a potential bias in this study. Among all participating units of the current study, the death rate in PICU from Hospital C was higher than others, mainly due to the fact that Hospital C is the largest transport center and receives very critical patients transferred from other hospitals. This may introduce some bias, which is a limitation of the study.

## Conclusion

Performances of the three scores in predicting PICU mortality are comparable, but less ideal than previous reports. Five predictors from the three score items were identified with better mortality prediction in Chinese PICU patients. Our findings provide important evidence for developing or updating the mortality prediction score for Chinese PICU patients.

## Data Availability Statement

The raw data supporting the conclusions of this article will be made available by the authors, without undue reservation.

## Ethics Statement

The studies involving human participants were reviewed and approved by Institutional Review Board of Children's Hospital of Fudan University. Written informed consent to participate in this study was provided by the participants' legal guardian/next of kin.

## Author Contributions

GL conceptualized and designed the study and reviewed and revised the manuscript. ZZ coordinated and supervised data collection, drafted the initial manuscript, and reviewed and revised the manuscript. WY and XH carried out the analyses, interpreted the results, drafted the initial manuscript, and reviewed and revised the manuscript. YW, YL, HoM, CZ, GP, YZ, XZ, WC, JL, DS, YB, ZC, BJ, HuM, XK, YChen, YCheng, and GY contributed to data collection and reviewed and revised the manuscript. All authors contributed to manuscript revision and read and approved the submitted version.

## Conflict of Interest

The authors declare that the research was conducted in the absence of any commercial or financial relationships that could be construed as a potential conflict of interest.

## Abbreviations

PCIS: pediatric critical illness score
PELOD: Pediatric Logistic Organ Dysfunction-2
PICU: pediatric intensive care unit
PIM: pediatric index of mortality
PRISM: pediatric risk of mortality.
