# Supplementary material for: Performance of Three Mortality Prediction Scores and Evaluation of Important Determinants in Eight Pediatric Intensive Care Units in China
Source: Front Pediatr. 2020 Sep 8;8:522. doi: 10.3389/fped.2020.00522 (PMC7505927; doi:10.3389/fped.2020.00522)
Supplement: Supplementary file 1 [file Data_Sheet_1.docx]

**Supplementary Table 1. Pediatric critical illness score (PCIS)**

**Supplementary Table 2. C-index of cox regression for three scores**

Supplementary Table 3. Results of Cox regression

**Supplementary Table 1. Pediatric critical illness score (PCIS)**

| variables | measurement | | Score |
| --- | --- | --- | --- |
|  | <1-year-old | ≥1-year-old |  |
| Heart Rate (beats per minute) | <80 or >160 | <60 or >160 | 4 |
|  | 80-100 or 160-180 | 60-80 or 140-160 | 6 |
| Other values |  |  | 10 |
| Systolic blood pressure kPa(mmHg) | <7.3 (55) or >17.3 (130) | <8.7 (65) or >20.0 (150) | 4 |
|  | 7.3-8.7 (55-65) or 13.3-17.3 (100-130) | 8.7-10 (65-75) or 17.3-20.0 (100-130) | 6 |
| Other values |  |  | 10 |
| Respiratory Rate (breaths per minute) | <20 or >70 or irregular respiratory rate | <15 or >60 or irregular respiratory rate | 4 |
|  | 20-25 or 40-70 | 15-20 or 35-60 | 6 |
| Other values |  |  | 10 |
| PaO2 kPa(mmHg) | <6.7 (50) | <6.7 (50) | 4 |
|  | 6.7-9.3 (50-70) | 6.7-9.3 (50-70) | 6 |
| Other values |  |  | 10 |
| PH | <7.25 or >7.55 | <7.25 or >7.55 | 4 |
|  | 7.25-7.3 or 7.5-7.55 | 7.25-7.3 or 7.5-7.55 | 6 |
| Other values |  |  | 10 |
| Sodium(mmol/L) | <120 or >160 | <120 or >160 | 4 |
|  | 120-130 or 150-160 | 120-130 or 150-160 | 6 |
| Other values |  |  | 10 |
| Potassium(mmol/L) | <3.0 or >6.5 | <3.0 or >6.5 | 4 |
|  | 3.0-.5 or 5.5-6.5 | 3.0-.5 or 5.5-6.5 | 6 |
| Other values |  |  | 10 |
| Creatinine(umol/L) | >159 | >159 | 4 |
|  | 106-159 | 106-159 | 6 |
| Other values |  |  | 10 |
| Blood Urea Nitrogen (mmol/L) | >14.3 | >14.3 | 4 |
|  | 7.1-14.3 | 7.1-14.3 | 6 |
| Other values |  |  | 10 |
| Hemoglobin (g/L) | <60 | <60 | 4 |
|  | <60-90 | <60-90 | 6 |
| Other values |  |  | 10 |
| Gastrointestinal | Stress ulcer bleeding and intestinal paralysis | Stress ulcer bleeding and intestinal paralysis | 4 |
|  | Stress ulcer bleeding | Stress ulcer bleeding | 6 |
| Other values |  |  | 10 |

**Supplementary Table 2. C-index of cox regression for three scores**

| **Scores** | **C-index** | **95% CI of hazard ratio** | |
| --- | --- | --- | --- |
|  |  | **Lower limit** | **Lower limit** |
| PRISM IV | 0.81 | 0.77 | 0.85 |
| PELOD-2 | 0.79 | 0.75 | 0.83 |
| PCIS^*^ | 0.77 | 0.73 | 0.81 |

*significantly lower than PRISM IV

Supplementary Table 3. Results of Cox regression

| **Predictors*** | **Hazard ratio** | **95% CI of hazard ratio** | | **P value** |
| --- | --- | --- | --- | --- |
|  |  | **Lower limit** | **Higher limit** |  |
| Invasive ventilation | 1.40 | 1.26 | 1.55 | <0.001 |
| Platelet | 1.85 | 1.59 | 2.16 | <0.001 |
| pH | 0.89 | 0.84 | 0.94 | <0.001 |
| Pupillary light reflex | 1.31 | 1.22 | 1.42 | <0.001 |
| PaO_2_ quantile^#^ |  |  |  |  |
| 1 | 2.60 | 1.61 | 4.19 | <0.001 |
| 2 | 1.46 | 0.88 | 2.44 | 0.146 |
| 3 | 1.92 | 1.15 | 3.22 | 0.013 |
| 5 | 1.36 | 0.79 | 2.33 | 0.261 |

* Invasive ventilation, platelet and pupillary light reflex scores are from PELOD-2, while pH and PaO_2_ scores are from PCIS.

# 4^th^ quantile as reference
